# Influence of environment on testing of hydraulic sealers

**DOI:** 10.1038/s41598-017-17280-7

**Published:** 2017-12-20

**Authors:** Mira Kebudi Benezra, Pierre Schembri Wismayer, Josette Camilleri

**Affiliations:** 1 Department of Endodontics, Faculty of Dentistry, Bezmialem Vakif University, Istanbul, Turkey; 2 Department of Anatomy, Faculty of Medicine and Surgery, University of Malta, Msida, Malta; 3 Department of Restorative Dentistry, Faculty of Dental Surgery, University of Malta, Msida, Malta; 4 School of Dentistry, University of Birmingham, Edgbaston, Birmingham, UK

## Abstract

*In vitro* material testing is undertaken by conducting a series of tests following procedures outlined in international standards. All material properties are measured in water; however biological behavior is undertaken in alternative media such as Dulbecco’s modified eagle medium (DMEM) or simulated body fluid. The aim of this study was to characterize four dental root canal sealers and study their properties in different media. Four dental root canal sealers were assessed. They were characterized by a combination of techniques and the sealer properties were tested as specified by ISO 6876 (2012) and also in alternative media. The sealer biocompatibility was measured by cell function and proliferation assays of elutions. All sealers complied with ISO specifications. The material properties were effected by the type of soaking medium used and the surface micromorphology and elemental composition were dependent on the soaking solution type. Both BioRoot and MTA Fillapex showed cytotoxicity which reduced at higher dilutions. The material chemistry, presentation, environmental conditions and testing methodology used affected the sealer properties. Standards specific to sealer type are thus indicated. Furthermore the methodology used in the standard testing should be more relevant to clinical situations.

## Introduction

A number of materials are used in various fields of dentistry. These materials should comply with the norms defined in international standards. Root canal sealer cements are used in obturation of root canals during root canal therapy. They are used in conjunction with solid gutta-percha points. The aim of using this material combination is the hermetic seal^1,2^ to avoid bacterial recontamination of the root canal space and thus treatment failure. The use of root canal sealers is mandatory to enhance the three dimensional compact sealing of gutta-percha in complex root canal systems. The properties of the ideal root canal sealer as suggested by Grossman^3^ include an excellent seal when set, dimensional stability, a slow setting time to ensure sufficient working time, insolubility to tissue fluids, adequate adhesion to canal walls, and biocompatibility. These sealers interact with the root dentine by mechanical interlocking and the formation of resin tags, which bind the sealer mechanically to the dentinal tubules^4–6^.

The conventional root canal sealer cements are classified according to the material chemistry and are tested following norms defined in ISO 6876; 2012^7^. During the last decade root canal sealers based on building material, Portland cement have been introduced and are known as hydraulic calcium silicate-based sealers which are also tested using the ISO 6876; 2012^7^. The popularity with these sealers is their hydraulic nature and their interaction with blood, tissue fluids and tooth tissue. Most of the properties of these materials depend on the formation of calcium hydroxide as a by-product of material hydration^8,9^. The release of this calcium hydroxide in solution is responsible for a number of properties that make this material popular for clinical use^10,11^. The release of the calcium hydroxide in solution renders the material soluble^12^ which in turn affects the other material properties such as biocompatibility, antimicrobial properties and physical and chemical characteristics^13,14^.

As indicated, the chemistry and material morphology of the different root canal sealers changes their physical, chemical and biological properties. When tested *in vitro* and when in use, the materials are subjected to different environments. The different environments have been shown to affect the material chemistry^15,16^ thus they can be postulated to also change the other material properties. The aim of this study was to characterise sealers based on tricalcium silicate, assess their properties according to ISO 6876; 2012^7^ specifications and re-evaluate if the sealer properties change in contact with different fluids used in biocompatibility testing and simulated body fluid. The cell proliferation and expression of the sealers was also evaluated.

## Results

### Material characterisation

**Figure 1:**
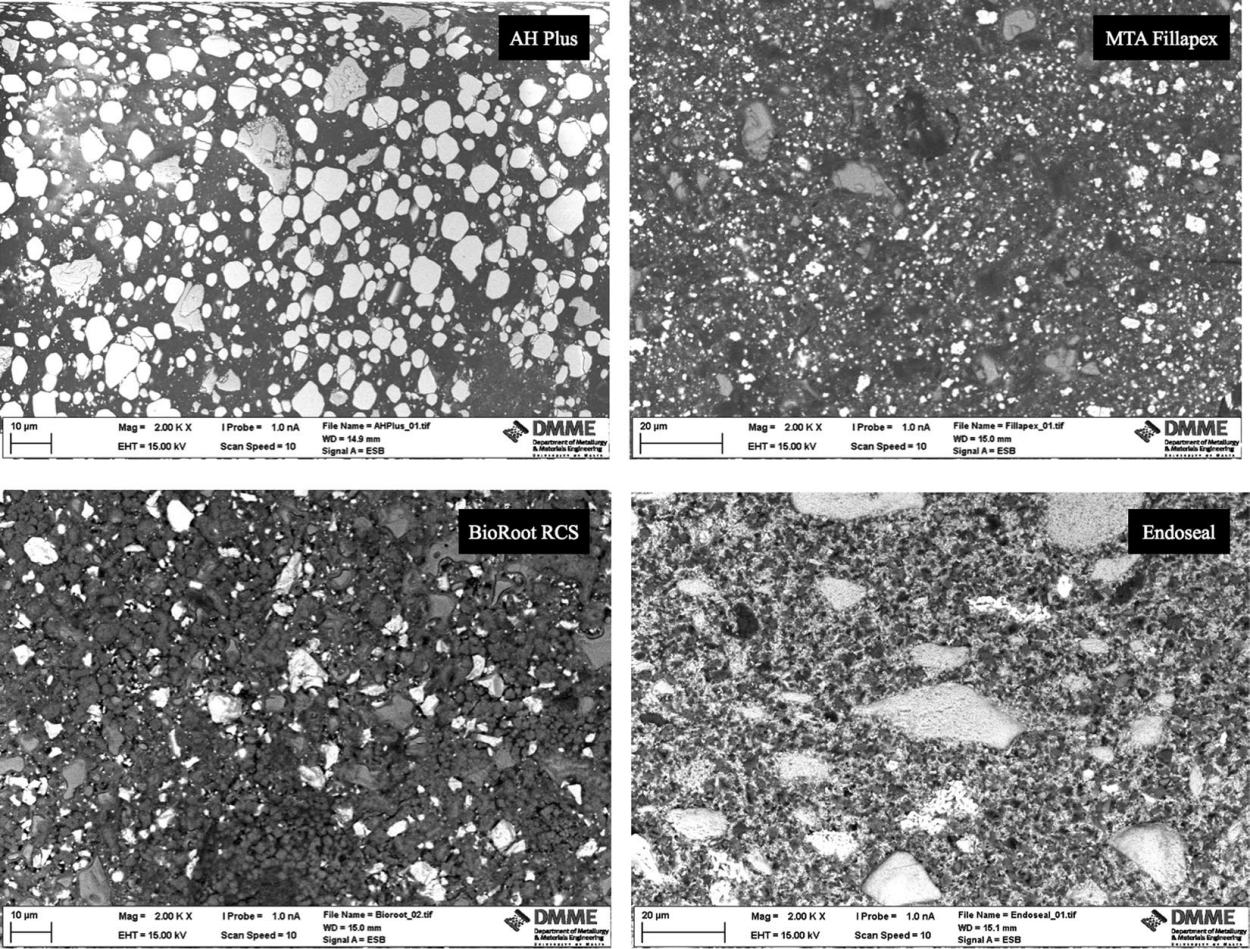
Back-scatter scanning electron micrographs of polished sections of test sealers showing micro-structural components.

**Figure 2:**
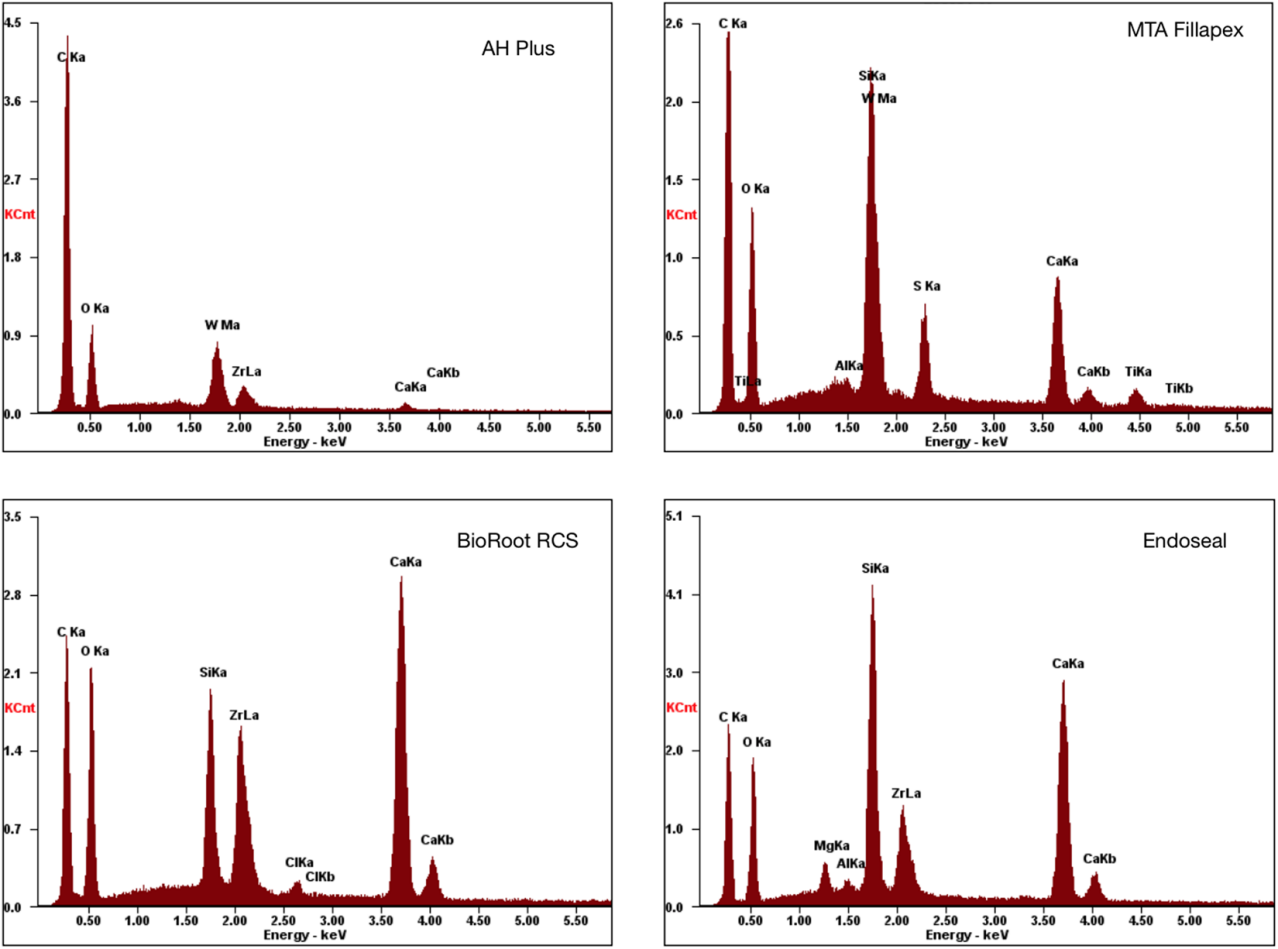
Energy dispersive spectroscopic analysis of the test materials showing the elemental analysis.

**Figure 3:**
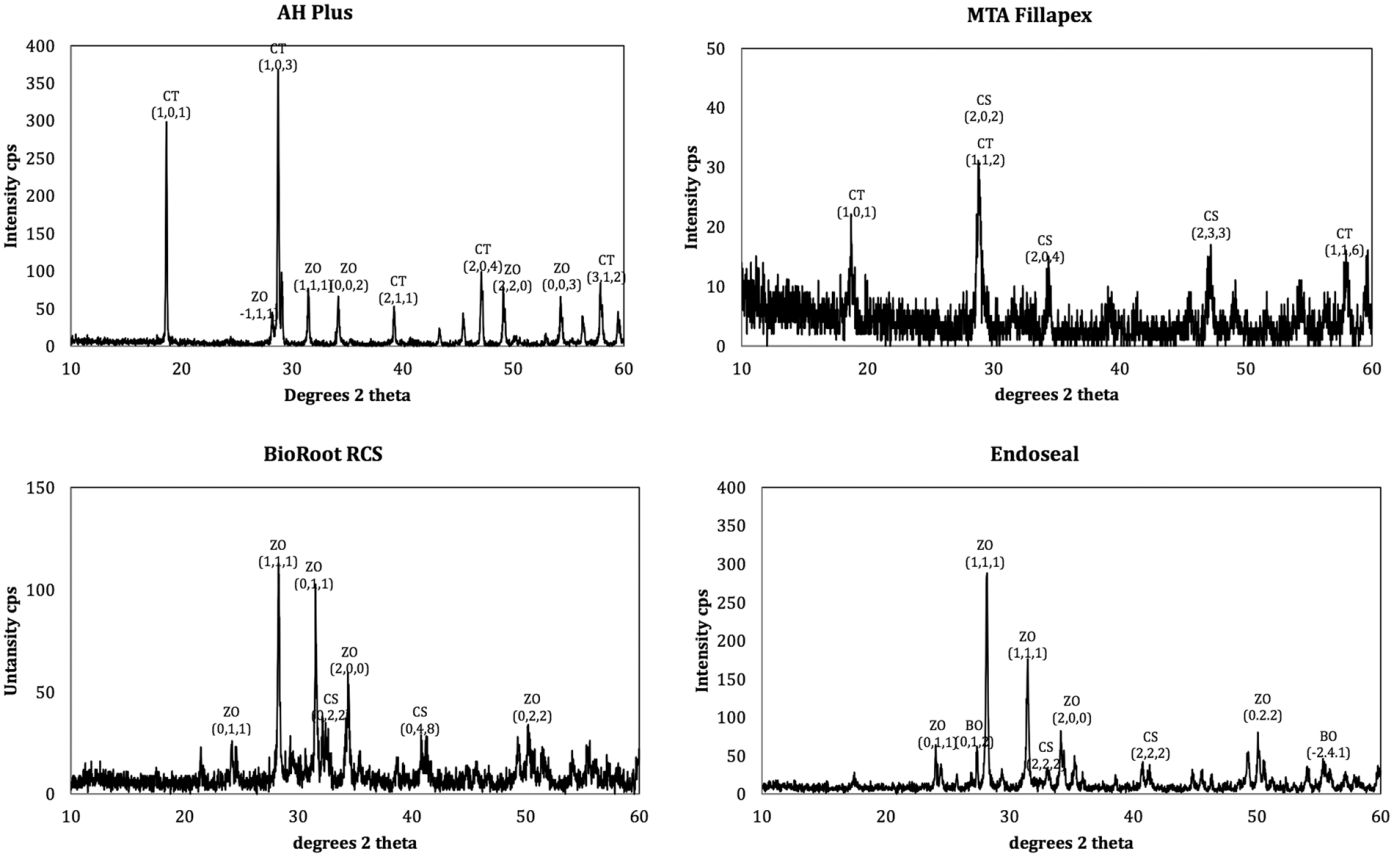
X-ray diffraction plots of test sealers showing the main crystalline phases present (BO: bismuth oxide, CS: calcium silicate, CT: calcium tungstate, ZO: zirconium oxide).

The scanning electron micrographs and EDS plots of all sealers tested are shown in Figs 1 and 2. The XRD plots in Fig. 3. Although the materials were all based on tricalcium silicate, the characterisation showed a different microstructure and presence of diverse radiopacifiers. The AH Plus included calcium tungstate (ICDD: 01-085-0443) and zirconium oxide (ICDD: 00-037-1484), the MTA Fillapex had calcium tungstate (ICDD: 00-041-1431), zirconium oxide in BioRoot RCS (ICDD: 04-015-6852) and zirconium oxide (ICDD: 04-015-4188) and bismuth oxide (ICDD: 04-003-2034) in Endoseal.

### Measurement of sealer physical properties

The results for the measurement of the physical properties described ISO 6876; 2012^7^ and the relevant modifications discussed in methodology are shown in Table 1.

**Table 1:** Results for testing of physical properties of test sealers in different environmental conditions ± SD.

Material	Media	Test			
		Flow	Film Thickness	Radiopacity	Setting Time
		*mm*	*μm*	*mm Al*	*mins*
AH Plus	as specified	24 ± 2	15 ± 5	16 ± 1	688
	HBSS	/	/	/	433
	DMEM	/	/	/	485
MTA Fillapex	as specified	30 ± 1	19 ± 4	4 ± 1	unset
	HBSS	/	/	/	unset
	DMEM	/	/	/	unset
BioRoot RCS	as specified	25 ± 1	46 ± 7	7 ± 1	235
	HBSS	/	/	/	667
	DMEM	/	/	/	650
Endoseal	as specified	25 ± 1	35 ± 5	12 ± 1	435
	HBSS	/	/	/	662
	DMEM	/	/	/	645
ISO Standard		6876	6876	6876	6876
value		>17	<50	<3	/

#### Flow and film thickness

The results for sealer flow, and film thickness are shown in Table 1 and compared to ISO norms. All sealers complied with the standards as they exhibited a flow greater than 17 mm and a film thickness smaller than 50 mm.

#### Radiopacity

All the sealers exhibited a radiopacity greater than 3 mm aluminium thickness (Table 1) specified by ISO 6876; 2012^7^.

#### Setting time

The MTA Fillapex did not set indefinitely. Although it was not completely unset an indentation could still be seen on the materials surface. Immersion in HBSS and DMEM did not affect the setting of this sealer (Table 1). The other tricalcium silicate-based sealers exhibited a lower setting time than AH Plus when allowed to set in air (P < 0.001) while immersion in HBSS and DMEM led to a longer setting time when compared to AH Plus (P < 0.001). Immersion in HBSS and DMEM reduced the setting time of AH Plus considerably (P < 0.001) while that of BioRoot RCS and Endoseal was extended when sealers were immersed in solution (Table 1).

#### Fluid uptake, sorption, solubility and porosity measurements

The results for the fluid uptake are shown in Table 2. The AH Plus exhibited the lowest fluid uptake of all the sealers tested. The solution type did not affect the fluid uptake of AH Plus which was low and increased slightly throughout the period of study. The BioRoot RCS exhibited slightly higher fluid uptake compared to AH Plus with a trend to increase in HBSS and DMEM. The one-day fluid uptake was high and reduced slightly after 7 days with an increase over the 28 day period. The Endoseal exhibited very high initial fluid uptake, which reduced over the 28-day testing period. The solution type did not affect the results obtained.

**Table 2:** Results for testing fluid uptake of test sealers in different environmental conditions ± SD.

Material	Media	TEST				
		Fluid Uptake (%)				
		*1 day*	*7 days*	*14 days*	*21 days*	*28 days*
AH Plus	water	0.2 ± 0.16	0.7 ± 0.21	1.1 ± 0.33	0.9 ± 0.18	2.2 ± 0.15
	HBSS	0.1 ± 0.05	0.5 ± 0.24	0.8 ± 0.30	0.5 ± 0.07	2.0 ± 0.15
	DMEM	0.3 ± 0.12	1.0 ± 0.23	0.9 ± 0.12	1.0 ± 0.37	2.2 ± 0.16
BioRoot RCS	water	2.4 ± 0.62	0.9 ± 1.62	1.2 ± 1.52	1.5 ± 1.37	5.5 ± 1.62
	HBSS	2.3 ± 0.54	1.2 ± 0.44	1.9 ± 0.83	2.1 ± 0.98	7.2 ± 0.71
	DMEM	1.7 ± 0.55	1.7 ± 1.48	2.3 ± 1.13	2.9 ± 1.05	9.8 ± 0.95
Endoseal	water	11.7 ± 2.14	1.4 ± 3.54	1.1 ± 3.94	1.1 ± 4.21	3.6 ± 3.92
	HBSS	10.5 ± 2.29	2.4 ± 1.96	1.9 ± 1.81	1.8 ± 1.78	4.5 ± 1.79
	DMEM	11.8 ± 1.58	1.3 ± 3.51	0.7 ± 3.22	0.7 ± 3.33	0.3 ± 3.33

*MTA Fillapex did not set so the fluid uptake could not be measured.

The MTA Fillapex could not be tested since it remained partially set. The sorption and solubility data shown in Table 3 shows that both properties for AH Plus were lower than that of the tricalcium silicate-based sealers for all the soaking solutions tested (P < 0.001). All the sealers tested complied to ISO 4049 recommendations^17^ for sorption except the Endoseal in DMEM after 28 days where the sorption was slightly higher than 40 mg/mm^3^. Since the ISO recommendations are for 1 day soaked materials this aberration was not considered to be significant. The solubility was high for both BioRoot RCS and Endoseal compared to ISO recommendations and increased over the 28-day period for all solutions tested. Using the ISO 6876 recommendations^7^ the solubility was mostly negative for all the sealers tested in HBSS and DMEM showing that rather than being soluble the sealers allowed deposition of matter on them thus increasing in weight showing a negative solubility values. This was very marked in AH Plus and Endoseal in DMEM when compared to the solubility of both sealers in water which is the liquid recommended for testing in ISO 6876^7^. These sealers are never in contact with water so results for solubility tested according to a specified standard are not significant *in vivo*

**Table 3:** Sorption and solubility values for test sealers using two standard methodologies (Mean ± SD).

Material	Media	Test				
		Sorption 28 days	Solubility 28 days	Sorption 1 day	Solubility 1 day	Solubility
		*mg/mm* ^*3*^	*mg/mm* ^*3*^	*mg/mm* ^*3*^	*mg/mm* ^*3*^	*%*
AH Plus	water	1.7 ± 0.4	0.3 ± 0.2	1.1 ± 0.1	0.6 ± 0.3	−0.04 ± 0.01
	HBSS	2.2 ± 1.0	0.4 ± 0.7	0.6 ± 0.1	0.4 ± 0.2	−2.3 ± 0.9
	DMEM	1.6 ± 0.6	0.4 ± 0.2	1.2 ± 0.3	0.4 ± 0.1	−25.1 ± 10.8
BioRoot	water	32.6 ± 1.8	36.1 ± 4.4	31.1 ± 2.9	26.6 ± 2.0	15.8 ± 5.7
	HBSS	34.1 ± 0.6	39.4 ± 1.9	33.5 ± 1.8	29.1 ± 0.9	30.2 ± 8.5
	DMEM	34.7 ± 3.2	41.4 ± 4.8	34.3 ± 0.6	31.1 ± 1.3	31.1 ± 4.3
Endoseal	water	39.3 ± 2.7	37.5 ± 9.9	40.4 ± 1.5	17.9 ± 4.4	1.4 ± 0.3
	HBSS	39.0 ± 2.7	35.9 ± 3.1	41.4 ± 3.4	20.0 ± 2.7	2.4 ± 1.7
	DMEM	44.1 ± 1.8	44.0 ± 6.6	38.5 ± 3.2	16.5 ± 5.2	20.4 ± 7.3
ISO Standard		4049	4049	4049	4049	6876
value		<40	<7.5	<40	<7.5	>3

*MTA Fillapex did not set so the fluid uptake could not be measured.

where the materials are in contact with physiological solution and more so in biological studies where the materials are placed in contact with DMEM and material solubility affects the results of testing. On the other hand the BioRoot RCS exhibited high solubility in water compared to the other sealers (P < 0.001) and negative solubility in both the HBSS and DMEM. The results of sealer solubility using different ISO recommendations were not comparable.

The results of the porosity of the test sealers calculated by gravimetric method after 1 and 28 day soaking in different solutions is show in Table 4. The AH Plus exhibited the lowest porosity at both 1 and 28 days in all media compared to the other sealers (P > 0.001). The BioRoot showed a ~2% porosity in the initial stages of setting and this value did not vary with the test medium. The porosity reduced to a negative value after 28 days. The Endoseal had the highest porosity after 1 day soaking which reduced considerably after 28 days (P < 0.001) for all media. The soaking media had no effect on the porosity values for all sealer types.

**Table 4:** Percentage porosity measured after 1 and 28 day immersion of test sealers in different media.

Material	Media	% Porosity	
		*1 day*	*28 days*
AH Plus	water	0.21 ± 0.16	0.73 ± 0.15
	HBSS	0.09 ± 0.05	0.68 ± 0.15
	DMEM	0.35 ± 0.12	0.73 ± 0.16
BioRoot RCS	water	2.42 ± 0.62	−1.82 ± 1.62
	HBSS	2.32 ± 0.54	−2.47 ± 0.71
	DMEM	1.70 ± 0.55	13.26 ± 0.95
Endoseal	water	11.69 ± 2.14	1.21 ± 3.92
	HBSS	10.54 ± 2.29	1.51 ± 1.79
	DMEM	11.85 ± 1.3	0.11 ± 3.33

*MTA Fillapex did not set so the percentage porosity could not be measured.

#### pH and chemical analyses of leachates

The results for measurement of pH and elemental analyses of soaking solutions over a period of 28 days are shown in Tables 5 and 6. All the sealers alkalinized the soaking solutions regardless the type of soaking solution used (Table 5). The pH rose over the 28-day period for the tricalcium silicate-based sealers while it decreased for AH Plus. The elemental analyses (Table 6) showed the same trends in both soaking solutions. Aluminium was leached in solution from both Endoseal and MTA Fillapex as both materials use cements which are based on Portland cement. Endoseal also leached bismuth in solution in higher quantities (P < 0.05) in DMEM than in HBSS. The other radiopacifiers were more stable particularly the zirconium where the release in solution was very low and independent on the soaking solution used (P > 0.05). The calcium ion release was highest in BioRoot RCS when compared to the other tricalcium silicate-based sealers (P < 0.05) and also the AH Plus (P < 0.001) which exhibited the lowest calcium release. The calcium ion release was higher in DMEM for all sealers (P < 0.05) except MTA Fillapex. Silicon was eluted in low quantities from all sealers except for MTA Fillapex; the release was independent of the solution used.

**Table 5:** pH values measured weekly over a 28 day period of test sealers in different media.

Material	Media	pH					
		*pH*	*1 day*	*7* *days*	*14* *days*	*21 days*	*28 days*
AH Plus	water	7.4	9.8 ± 0.2	9.2 ± 0.1	9.1 ± 0.3	9.4 ± 0.3	9.8 ± 0.4
	HBSS	8.5	10.5 ± 0.2	7.5 ± 0.2	7.6 ± 0.3	7.5 ± 0.2	7.6 ± 0.2
	DMEM	7.9	10.5 ± 0.2	8.3 ± 0.5	8.4 ± 0.5	8.4 ± 0.1	6.1 ± 0.4
MTA Fillapex	water	7.4	10.4 ± 0.1	11 ± 0.1	10.4 ± 0.1	10.4 ± 0.0	9.8 ± 0.1
	HBSS	8.5	10 ± 0.1	10.5 ± 0.1	10.2 ± 0.1	10.2 ± 0.1	9.8 ± 0.0
	DMEM	7.9	8.5 ± 0.2	10.2 ± 0.1	10 ± 0.1	10 ± 0.1	9.6 ± 0.1
BioRoot RCS	water	7.4	10.5 ± 0.05	12.5 ± 0.1	12.4 ± 0.1	12.4 ± 0.1	12.4 ± 0.0
	HBSS	8.5	10.5 ± 0.05	12.1 ± 0.5	12.4 ± 0.1	12.3 ± 0.2	12.5 ± 0.0
	DMEM	7.9	10.5 ± 0.1	12.4 ± 0.1	12.5 ± 0.1	12.4 ± 0.1	12.5 ± 0.1
Endoseal	water	7.4	10.5 ± 0.1	12.2 ± 0.1	11.9 ± 0.2	12.2 ± 0.1	12.2 ± 0.1
	HBSS	8.5	10.9 ± 0.2	11.5 ± 0.1	11.6 ± 0.1	11.8 ± 0.1	11.7 ± 0.1
	DMEM	7.9	10.5 ± 0.3	10.7 ± 0.2	10.6 ± 0.1	10.9 ± 0.1	10.9 ± 0.1

**Table 6:** Elements detected in leachate from different sealers after 28 day exposure to different media.

Material	Media	Elements detected in leachate mg/L						
		Al	Bi	Ca	P	Si	W	Zr
AH Plus	HBSS	BDL	BDL	2.27	26.23	0.48	4.64	0.01
	DMEM	BDL	BDL	37.18	27.05	0.83	0.09	BDL
MTA Fillapex	HBSS	0.02	BDL	441.50	BDL	91.78	0.07	BDL
	DMEM	0.02	BDL	442.80	2.06	82.70	0.09	BDL
BioRoot RCS	HBSS	BDL	BDL	1533.00	0.79	0.52	BDL	BDL
	DMEM	BDL	BDL	1709.00	2.87	0.29	BDL	0.01
Endoseal	HBSS	8.90	0.39	264.80	0.12	3.94	BDL	0.03
	DMEM	5.82	10.30	636.90	23.66	7.36	BDL	0.04

BDL: below detection limit.

### Investigation of biological activity

**Figure 4:**
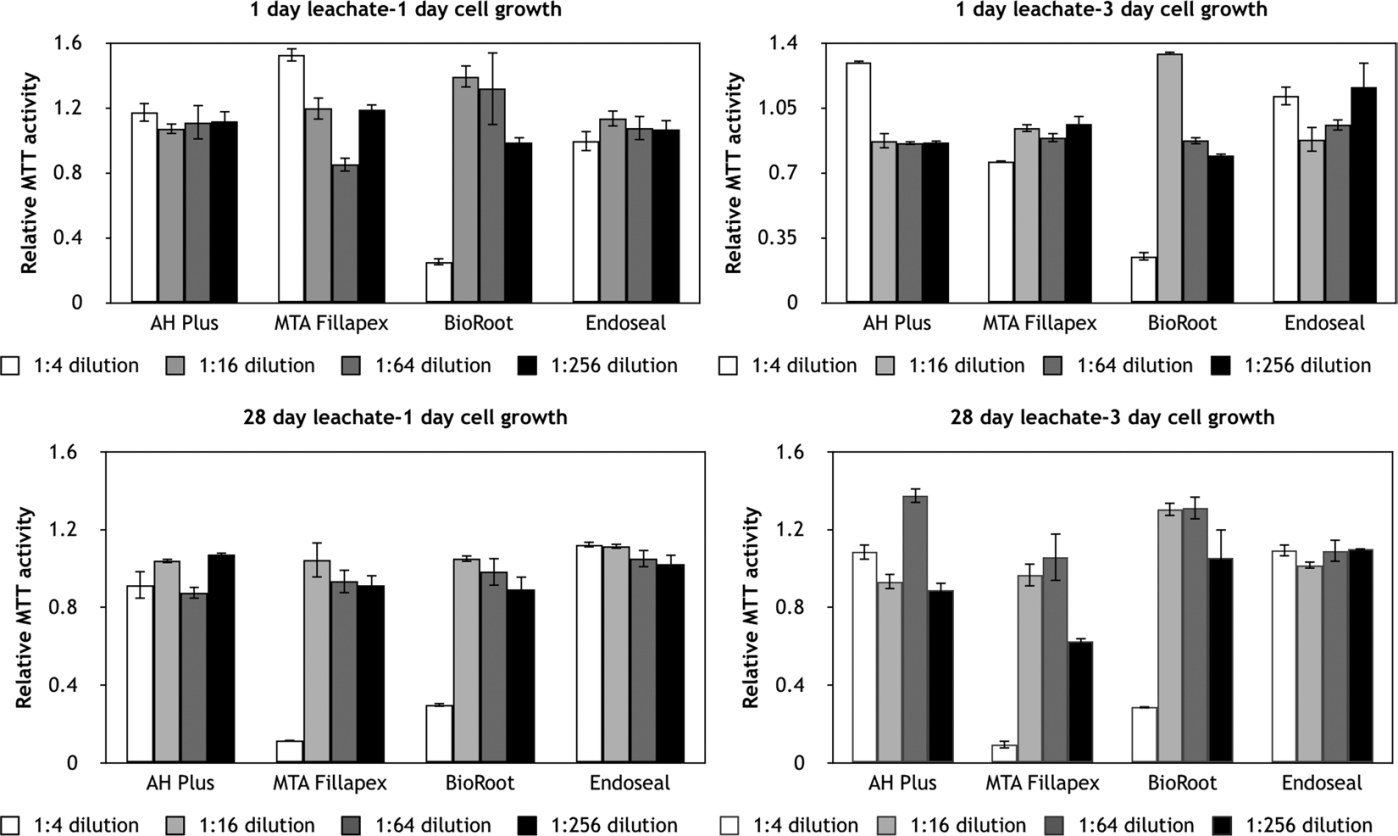
Cell proliferation and expression of gingival fibroblasts in response to exposure of leachates at different dilutions from test sealers in indirect contact test.

The results for the indirect contact test are shown in Fig. 4. The BioRoot RCS leachate was toxic to the gingival fibroblasts both in the 1-day and the 28-day leachates with cell activity enhanced after 3-days of exposure. This effect was reduced at higher dilutions. The same was observed for MTA Fillapex in the 28 day leachate. The MTA Fillapex exhibited optimal cell activity in the 1-day leachate, which deteriorated in the 28-day leachate. Cell activity improved over the 3-day exposure of the 28-day leachate. This suggests that MTA Fillapex initially does not leach anything toxic but over a longer time, other chemicals leach out which, have a more toxic or inhibitory effect on cell growth. This is confirmed by the enhanced cell activity at higher dilutions. The AH Plus and Endoseal showed stable cell activity in both 1 and 28-day leachates after both exposure times.

### Surface characterization of materials after contact with different solutions

**Figure 5:**
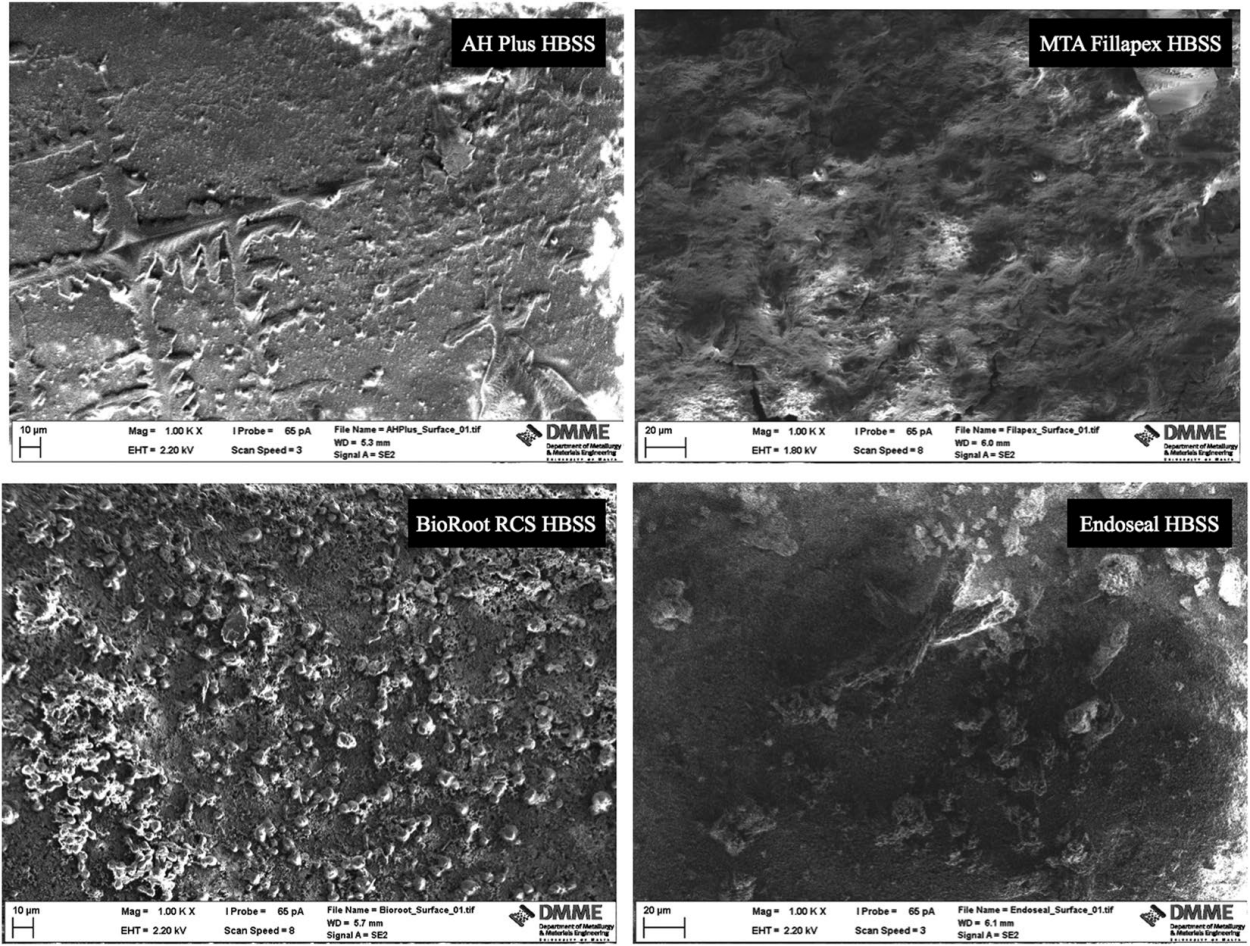
Secondary electron scanning electron micrographs of sealers immersed in HBSS to assess surface microstructure.

**Figure 6:**
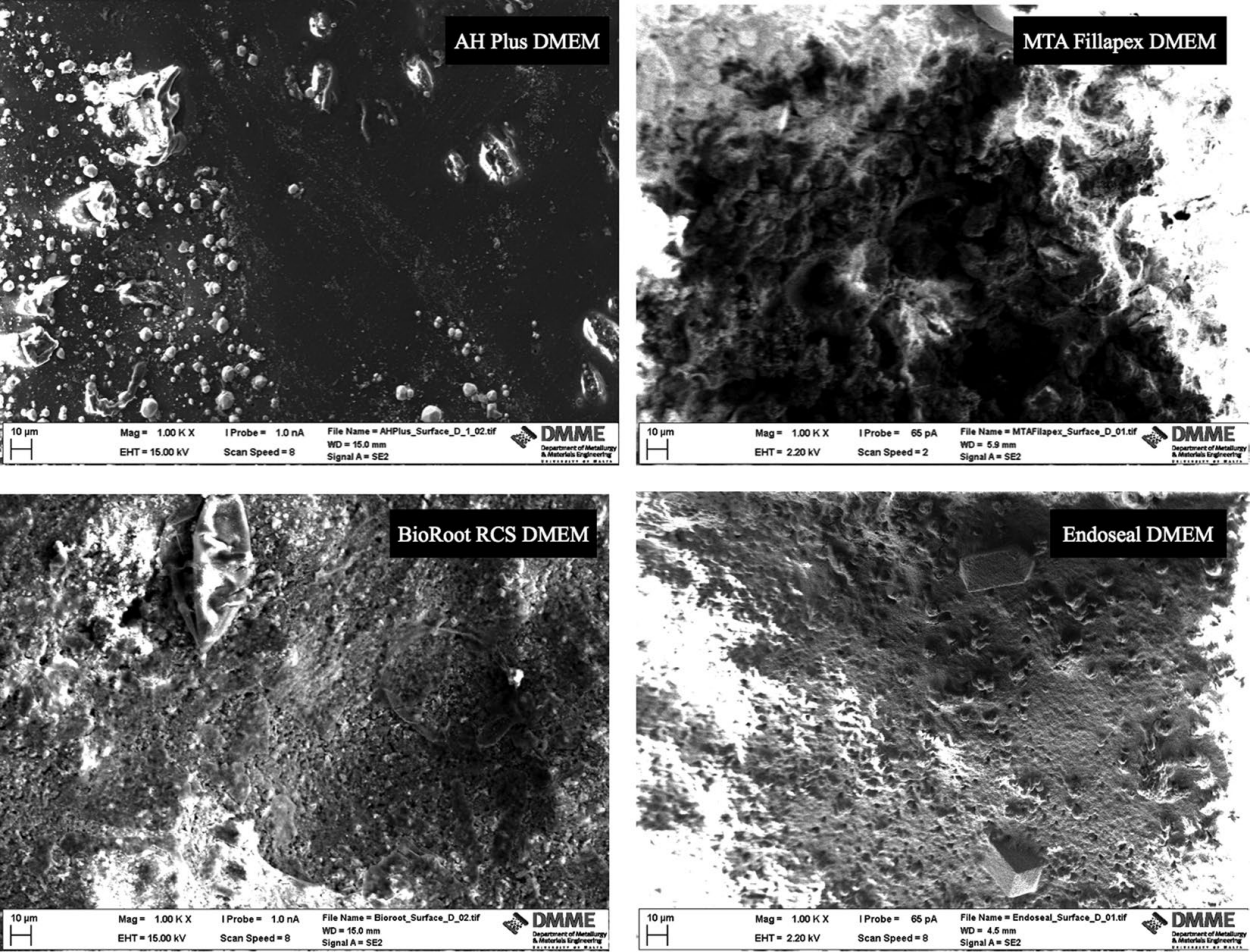
Secondary electron scanning electron micrographs of sealers immersed in DMEM to assess surface microstructure.

**Figure 7:**
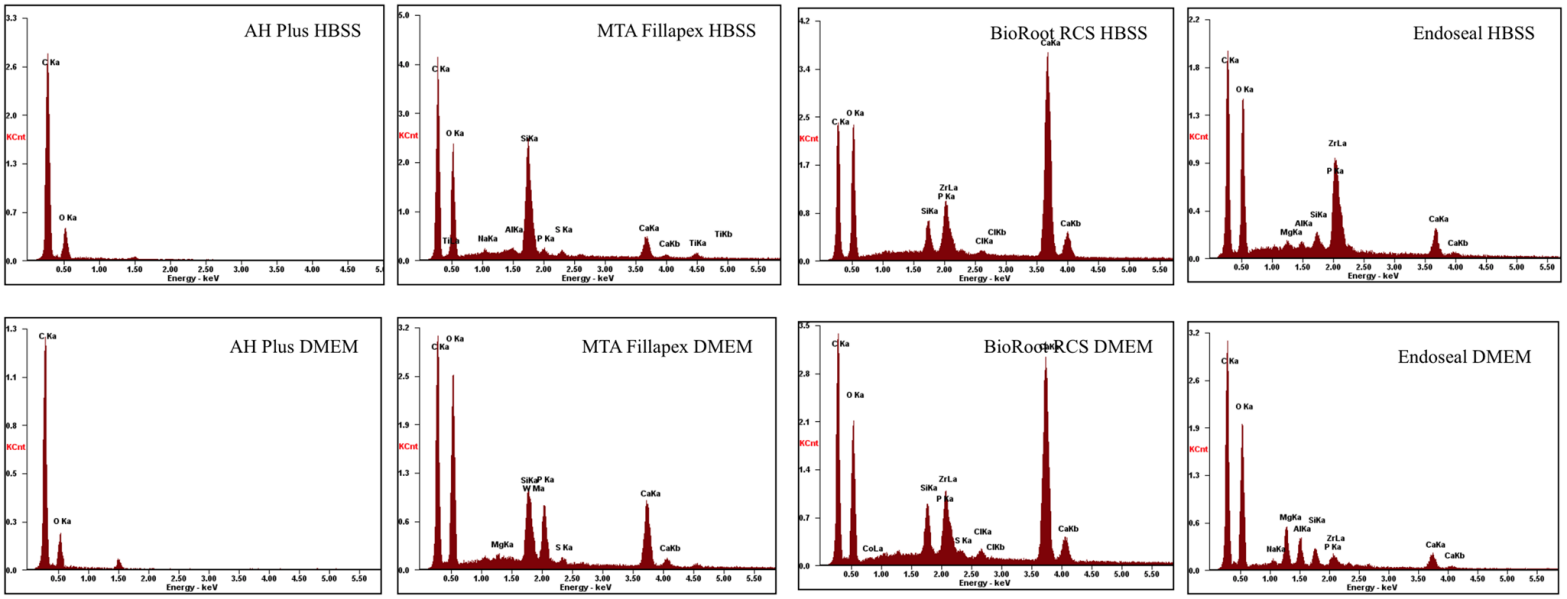
Energy dispersive spectroscopic scans of sealer surfaces in contact with different solutions.

The surface morphology and elemental analyses of the materials in contact with either DMEM or HBSS is shown in Figs 5–7. This test was performed in order to evaluate the surface morphology of the materials in contact with DMEM (Fig. 6) and whether the surface morphology varies compared to that in contact with HBSS (Fig. 5), which is usually used to evaluate the material bioactivity. The sealer surfaces in contact with DMEM were different to those in contact with HBSS for all sealer types except AH Plus (Figs 5 and 6). The P peak in relation to the Ca peak was higher in DMEM for MTA Fillapex and BioRoot RCS as opposed to the Endoseal where a higher P peak was observed in HBSS (Fig. 7).

## Discussion

The current study investigated four sealer types. The sealers were based on tricalcium silicate and thus leach calcium hydroxide making them susceptible to reaction with environmental fluids. AH Plus was used as a control sealer. The MTA Fillapex used in this study was a new version, which was recently launched by Angelus. It is bismuth oxide-free to avoid tooth discoloration as bismuth oxide containing materials were shown to discolour teeth when in contact with sodium hypochlorite which is used in all root canal treatments^18^. The composition of BioRoot RCS was in accordance with previous studies^19,20^. Endoseal is also a relatively new sealer on the market thus not characterized. A combination of scanning electron microscopy, energy dispersive spectroscopy and X-ray diffraction analyses was used to characterize the sealers.

The differences in the material composition and presentation were correlated to the material properties and their behaviour when exposed to different environments. In fact the leaching of calcium ions in solution was less for Endoseal than for BioRoot RCS as indicated in the leachate analyses. BioRoot RCS was shown in previous studies to leach high levels of calcium compared to other tricalcium silicate-based endodontic cements^19^.

All sealers complied with ISO 6876; 2012^7^ standards for flow, film thickness and radiopacity. These tests were run following the ISO recommendations. The testing of setting time, fluid uptake, sorption, solubility and porosity were conducted in air/water as suggested by the ISO standards but also in DMEM and HBSS. A number of material properties such as biocompatibility and bioactivity depend on these properties. The HBSS is used to simulate *in vivo* conditions while DMEM is used in cytology. Variations of material properties in contact with these fluids can affect the related biological characteristics. In addition where applicable different standards were used and the results obtained compared.

The BioRoot RCS exhibited high fluid uptake, which reduced over the 28-day period and was dependent on the soaking solution as opposed to the Endoseal where the solution type did not affect the fluid uptake. The high solubility exhibited by BioRoot RCS and the different solubility demonstrated in different soaking media has been reported^12^. The high solubility observed for Endoseal in the current study has already been reported^21^. The solubility was high for both BioRoot RCS and Endoseal using the gravimetric method and the formulae suggested in ISO 4049^17^. The results obtained for both materials using the ISO 6876^7^ were different. Thus standards specific to the material type are necessary particularly for tricalcium silicate-based materials, which possess particular and distinguishing properties when compared to other sealer types.

The leaching in DMEM was higher than in HBSS for most elements. The leaching depends on the material solubility and solubility was also shown to be dependent on the soaking solution. The MTA Fillapex leachate was shown to enhance cell attachment and proliferation. The BioRoot RCS in comparison was shown to be cytotoxic but the cell growth resumed at higher dilutions. This is in contrast to previous research showing BioRoot RCS to be biocompatible tested using periodontal ligament stem cells^22^. Furthermore the BioRoot RCS was shown to enhance the stem cells better than the Endoseal also in contrast to the findings in the current study. Previous research on biocompatibility of Endoseal implanted in subcutaneous tissues of rats showed Endoseal to have a similar reaction to MTA and better than AH Plus^21^. This is also inferred in the current study at the cellular level. Furthermore Endoseal was shown to enhance cell activity better than MTA Fillapex^23^. However the data cannot be compared to the current study since the MTA Fillapex used in the previous research may have been the bismuth-containing MTA Fillapex. Material characterization is necessary in every research work to make sure that the materials are well characterized to enable comparison to further research.

The energy dispersive spectroscopic data of the sealer surfaces after exposure to the DMEM and HBSS indicate that the material chemistry changes and the surface morphology as well. Thus data obtained after exposure to simulated body fluid cannot be extrapolated for cytology where DMEM is used.

## Methods

The following root canal sealers were used in this study:

1. AH Plus (Dentsply, DeTrey GmbH, Konstanz, Germany)
2. MTA Fillapex (Angelus, Londrina, Brazil)
3. BioRoot RCS (Septodont, Saint-Maur-des-Fossés, France)
4. Endoseal (Maruchi, Wonju-si, Gangwon-do, South Korea)

The composition of the sealers as provided by the manufacturers is shown in Table 7. All sealers were mixed and manipulated in accordance with the manufacturers’ instructions, except for Endoseal, a premixed root canal sealer that was syringed. The environmental factors were modified as indicated below. The sealers were tested according to the standard specifications but in addition were also immersed in Hank’s balanced salt solution (HBSS, Sigma Aldrich, Gillingham UK) and Dulbecco’s modified eagle medium (DMEM; Sigma Aldrich, Gillingham UK). This was done in order to investigate the material properties in contact with simulated tissue fluids and fluids used for cell culture.

**Table 7:** Constituents of sealers tested.

Name	Presentation	Chemical composition	
		*Component 1*	*Component 2*
AH Plus	Two tubes	Diepoxide, calcium tungstate, zirconium oxide, aerosil, pigment	1-adamantane amine N,N′-dibenzyl-5-oxa-nonandiamine-1,9 TCD-Diamine, calcium tungstate, zirconium oxide, aerosil, silicone oil
MTA Fillapex	Two tubes	Methyl salicylate, butylene glycol, colophony, calcium tungstate, silicon oxide	Mineral trioxide aggregate, silicon dioxide, titanium dioxide, pentaerythritol, rosinate, P - Toluenesolfonamide
BioRoot	Powder/liquid	Tricalcium silicate, zirconium oxide	Water, calcium chloride, water-soluble polymer
Endoseal	1 tube	Calcium silicates, calcium aluminate, calcium aluminoferrite, calcium sulphate, radiopacifier, thickening agent	/

### Material characterization

The set sealers were characterized by scanning electron microscopy (SEM) and energy dispersive spectroscopy (EDS) and X-ray diffraction analysis (XRD). The micromorphology at different magnifications was assessed on polished specimens using a scanning electron microscope (SEM; Zeiss MERLIN Field Emission SEM, Carl Zeiss NTS GmbH, Oberkochen, Germany). Phase analysis was performed on powdered sealers after 1 or 28 day immersion in HBSS using a Bruker D8 diffractometer (Bruker Corp., Billerica, MA, USA) with Co Kα radiation (1.78 A°). The X-ray patterns were acquired in the 2θ (10–60°) with a step of 0.02° and 0.5 seconds per step. Phase identification was accomplished using a search-match software utilizing ICDD database (International Centre for Diffraction Data, Newtown Square, PA, USA).

### Assessment of physical and chemical properties

#### Assessment of film thickness, flow, setting time and radiopacity

Film thickness, sealer flow, setting time and radiopacity were assessed following ISO 6876; 2012^7^. For setting time the testing was performed also with material immersed in HBSS and DMEM.

#### Investigation of fluid uptake, sorption, solubility and porosity

Fluid uptake, sorption and solubility was performed as specified in ISO 4049; 2009 using water and also Hank’s balanced salt solution (HBSS; Sigma Aldrich, St Louis, MO, USA) and Dulbecco’s modified eagle medium (DMEM). Weight changes were recorded after 7, 14, 21 and 28 days thus enabling the calculation of fluid uptake at each specified interval. Sorption and solubility were also calculated. In addition the sealer solubility was also assessed using ISO 6876; 2012^7^ procedure with water, HBSS and DMEM media used to soak the different sealers.

Porosity was assessed by calculating the porosity for the materials using a gravimetric method as described in Cutajar *et al*.; 2011^24^ after 1 and 28 day-immersion in different solutions namely water, HBSS and DMEM.

#### Assessment of pH and chemistry of leachates

The pH of the soaking solutions before and after immersion (7, 14, 21 and 28 days) of the test sealers was measured with a pH meter (Hanna HI 3221, Hanna Instruments, Woonsocket, RI, USA). For leachate analysis, cylindrical specimens (10 mm in diameter and 2 mm thick) were prepared. They were allowed to set for 24 hours at 37 ± 1 °C, weighed, after which the materials were immersed in 5 mL of HBSS or DMEM at 37 ± 1 °C for 28 days. The sealers were removed from the storage solution and discarded. The storage solution and a blank were assessed using inductively coupled plasma (ICP).

### Investigation of sealer biological properties

The cytocompatibility of the test materials was evaluated *in vitro* on human gingival fibroblasts following to ISO 10993-5;2009^25^ using an indirect testing method. The 3-(4,5 dimethylthiazolyl-2-yl)-2,5-diphenyltetrazolium bromide (MTT) assay^26^ was used to assess cell metabolic function. The leachate extraction was made in cell culture medium without serum and antibiotics using a surface area (sample) to volume (medium) ratio of approximately 150 mm^2^/ml. 24 hours after setting the sealers were exposed to medium for either 1 day or 28 days. The extract collected at each time point was serially diluted to 1:2, 1:8, 1:32 and 1:128 with fresh DMEM. DMEM alone served as a negative control. Cells were seeded in 96-well plates at 1.5 × 10^4^ cells/well in 100 μl of DMEM. Five repeats were performed for every solution. After overnight attachment, cells were treated with various extracts of sealers (100 μl /well) resulting in final concentrations of the sealer-conditioned medium of 1:4, 1:16, 1:64 and 1:256.

### Surface characterization of materials after contact with different solutions

In addition, the sealer surfaces in contact with different media were assessed by scanning electron microscopy and energy dispersive spectroscopy in order to evaluate the effect of the media used on sealer chemistry and surface morphology after immersion in HBSS or DMEM for 28 days.

### Statistical Analysis

The data were evaluated using Statistical Package for the Social Sciences software (PASW Statistics 18; SPSS Inc, Chicago, IL). One-way analysis of variance and Tukey post-hoc tests at a significance level of P = 0.05 were used to perform multiple comparison tests.

## Conclusions

The material chemistry, presentation, environmental conditions and testing methodology used affected the sealer properties. Standards specific to sealer type are thus indicated. Furthermore the methodology used in the standard testing should be more relevant to clinical situations.
