# Author Correction: Influence of environment on testing of hydraulic sealers

**DOI:** 10.1038/s41598-018-24847-5

**Published:** 2018-05-03

**Authors:** Mira Kebudi Benezra, Pierre Schembri Wismayer, Josette Camilleri

**Affiliations:** 1 Department of Endodontics, Faculty of Dentistry, Bezmialem Vakif University, Istanbul, Turkey; 2 Department of Anatomy, Faculty of Medicine and Surgery, University of Malta, Msida, Malta; 3 Department of Restorative Dentistry, Faculty of Dental Surgery, University of Malta, Msida, Malta; 4 School of Dentistry, University of Birmingham, Edgbaston, Birmingham UK

Correction to: *Scientific Reports* 10.1038/s41598-017-17280-7, published online 20 December 2017

In Table 3 of the Article the units µg/mm^3^ are incorrectly given as mg/mm^3^ and some negative values are incorrectly given as positive in the columns ‘Solubility 28 Days’ and ‘Solubility’. In addition, the Solubility % value ‘<3’ is incorrectly given as ‘>3’. The correct Table 3 appears below.

**Table 3:** Sorption and solubility values for test sealers using two standard methodologies (Mean ± SD).

Material	Media	Test				
		Sorption 28 days	Solubility 28 days	Sorption 1 day	Solubility 1 day	Solubility
		*μg*/*mm*^*3*^	*μg*/*mm*^*3*^	*μg*/*mm*^*3*^	*μg*/*mm*^*3*^	%
AH Plus	water	1.7 ± 0.4	−0.3 ± 0.2	1.1 ± 0.1	0.6 ± 0.3	−0.04 ± 0.01
	HBSS	2.2 ± 1.0	0.4 ± 0.7	0.6 ± 0.1	0.4 ± 0.2	−2.3 ± 0.9
	DMEM	1.6 ± 0.6	−0.4 ± 0.2	1.2 ± 0.3	0.4 ± 0.1	−25.1 ± 10.8
BioRoot	water	32.6 ± 1.8	36.1 ± 4.4	31.1 ± 2.9	26.6 ± 2.0	15.8 ± 5.7
	HBSS	34.1 ± 0.6	39.4 ± 1.9	33.5 ± 1.8	29.1 ± 0.9	−30.2 ± 8.5
	DMEM	34.7 ± 3.2	41.4 ± 4.8	34.3 ± 0.6	31.1 ± 1.3	−31.1 ± 4.3
Endoseal	water	39.3 ± 2.7	37.5 ± 9.9	40.4 ± 1.5	17.9 ± 4.4	1.4 ± 0.3
	HBSS	39.0 ± 2.7	35.9 ± 3.1	41.4 ± 3.4	20.0 ± 2.7	−2.4 ± 1.7
	DMEM	44.1 ± 1.8	44.0 ± 6.6	38.5 ± 3.2	16.5 ± 5.2	−20.4 ± 7.3
ISO Standard		4049	4049	4049	4049	6876
value		<40	<7.5	<40	<7.5	<3

*MTA Fillapex did not set so the fluid uptake could not be measured.

